# Tofacitinib Regulates Endostatin via Effects on CD147 and Cathepsin S

**DOI:** 10.3390/ijms25137267

**Published:** 2024-07-02

**Authors:** Devy Zisman, Hala Sabtan, Maya M. Rahat, Elina Simanovich, Amir Haddad, Tal Gazitt, Joy Feld, Gleb Slobodin, Adi Kibari, Muna Elias, Michal A. Rahat

**Affiliations:** 1 Department of Rheumatology, Carmel Medical Center, Haifa 3436212, Israel; 2 Rappaport Faculty of Medicine, Technion-Israel Institute of Technology, Haifa 3525422, Israel; 3 Immunotherapy Laboratory, Carmel Medical Center, Haifa 3436212, Israel; 4 Rheumatology Unit, Bnai Zion Medical Center, Haifa 3339419, Israel

**Keywords:** tofacitinib, endostatin, CD147/EMMPRIN, rheumatoid arthritis (RA), angiogenesis, STAT3, miR-146a-5p 1

## Abstract

Angiogenesis is critical for rheumatoid arthritis (RA) progression. The effects of tofacitinib, a JAK-STAT inhibitor used for RA treatment, on angiogenesis in RA are unclear. We, therefore, evaluated the levels of angiogenic factors in two systems of a human co-culture of fibroblast (HT1080) and monocytic (U937) cell lines treated with tofacitinib and in serum samples from RA patients before and after six months of tofacitinib treatment. Tofacitinib reduced CD147 levels, matrix metalloproteinase-9 (MMP-9) activity, and angiogenic potential but increased endostatin levels and secreted proteasome 20S activity. In vitro, tofacitinib did not change CD147 mRNA but increased miR-146a-5p expression and reduced STAT3 phosphorylation. We recently showed that CD147 regulates the ability of MMP-9 and secreted proteasome 20S to cleave collagen XVIIIA into endostatin. We show here that tofacitinib-enhanced endostatin levels are mediated by CD147, as CD147-siRNA or an anti-CD147 antibody blocked proteasome 20S activity. The correlation between CD147 and different disease severity scores supported this role. Lastly, tofacitinib reduced endostatin’ s degradation by inhibiting cathepsin S activity and recombinant cathepsin S reversed this in both systems. Thus, tofacitinib inhibits angiogenesis by reducing pro-angiogenic factors and enhancing the anti-angiogenic factor endostatin in a dual effect mediated partly through CD147 and partly through cathepsin S.

## 1. Introduction

Rheumatoid arthritis (RA) is an autoimmune chronic inflammatory disease, affecting mainly synovial joints, with increased leukocyte infiltration and synovial hyper-proliferation, resulting in swelling of the joints, pain and stiffness, and gradual and ongoing tissue damage and bone erosion. In addition, extra-articular manifestations, such as lungs, eyes, heart, and skin, as well as comorbidities, especially with cardiovascular involvement, have a significant impact on life quality, morbidity, and mortality [1]. The increased number of cells in the synovium and their enhanced metabolic need for oxygen and nutrients result in local hypoxia, which strongly drives angiogenesis [2]. Additionally, cytokines (e.g., TNFα, IL-6), proteases (especially matrix metalloproteinases (MMPs)), and growth factors (e.g., VEGF, TGFβ) locally secreted by synoviocytes and infiltrating leukocytes also have pro-angiogenic activities [3]. Angiogenesis is the process of new blood vessels sprouting from existing ones. It is regulated by the fine balance between pro-angiogenic factors (e.g., vascular endothelial growth factor (VEGF), matrix metalloproteinases (MMPs)) that promote the activation and proliferation of endothelial cells and their migration through the extracellular matrix (ECM) [4,5] and anti-angiogenic factors (e.g., endostatin, thrombospondin-1 (Tsp-1)) that inhibit their activation and migration [6,7]. An increase in the concentrations of the pro-angiogenic factors over the anti-angiogenic factors disrupts this balance and induces angiogenesis [8].

One of the important pro-angiogenic factors is the extracellular matrix metalloproteinase inducer (EMMPRIN/CD147), a trans-membranal and secreted protein with multiple functions that depend on the proteins it interacts with [9]. CD147 is not only a pro-inflammatory factor that is expressed on macrophages, T cells, fibroblasts, and endothelial cells, but primarily it is considered a pro-angiogenic factor due to its ability to induce the production of MMPs and VEGF [10,11] and to enhance the expression of VEGF receptor II on endothelial cells [12]. We have previously described its role, including in a secreted form, in enhancing angiogenesis in an in vitro co-culture system of human monocytic and fibroblastic cell lines, as well as in RA and psoriatic arthritis patients [13,14]. However, the regulation of CD147 expression is not well understood.

Endostatin is a potent anti-angiogenic factor that can bind to several receptors, such as VEGFR2, integrins, glypican-1, and glypican-4, to inhibit their downstream activation of ERK and p38 MAPKs [6,15]. Endostatin is the product of the proteolytic cleavage of the C-terminus domain of collagen XVIIIA (Col18A). Several proteases, including cathepsin S, cathepsin L, MMP-7, MMP-9, MMP-14, elastin, and secreted proteasome 20S, can cleave this domain into endostatin or into fragments with different molecular weights that include endostatin [16,17,18,19]. The final 22 kDa cleavage product endostatin is found in the basement membrane and ECM, deposited by many types of cells [20]. We have recently demonstrated that soluble CD147 can regulate both MMP-9 and secreted proteasome 20S that cooperate to cleave Col18A and generate endostatin [21].

The treatment of RA is aimed to achieve disease remission or low disease activity and is based on treatment with non-steroidal anti-inflammatory drugs or glucocorticosteroids to ameliorate symptoms and treatment with disease-modifying anti-rheumatic drugs (DMARDs). First-line treatment consists of conventional synthetic DMARDs (csDMARDs), such as methotrexate (MTX), leflunomide, and sulfasalazine. If the treatment goal is not achieved, biological DMARDs (bDMARDs), such as tumor necrosis factor inhibitors, IL-6 inhibitors, anti-CD20 or anti-CTL4, or targeted synthetic DMARDs (tsDMARDs), such as the Janus kinase inhibitors tofacitinib, baricitinib, filgotinib, or upadacitinib, are added to the treatment regimen [22].

Tofacitinib was the first JAK inhibitor to be approved by the FDA to treat RA patients. Tofacitinib is an oral small-molecule inhibitor, which belongs to a family of JAKinibs that inhibit the JAK family of proteins. It selectively inhibits JAK3 and, to a lesser degree, JAK2 and JAK1 and, therefore, inhibits the JAK–STAT pathway [23,24]. Whereas JAK3 is triggered by a small number of cytokine receptors (e.g., IL-2, IL-4, IL-7, IL-9, IL-15, and IL-21), JAK1 and JAK2 are crucial for the signaling of many cytokine receptors [25]. Thus, the ability to inhibit the signaling of many cytokines promotes the overall ability of tofacitinib to inhibit the proliferation, inflammation, and migration of cells [23,26]. In addition to their ability to mediate inflammation, cytokines that trigger the JAK–STAT pathways are also drivers of angiogenesis, particularly through STAT3 [27]. Therefore, the JAK inhibitors, including tofacitinib, are likely to inhibit angiogenesis as well. However, this aspect of their activity has been hardly explored and the effects of tofacitinib on angiogenesis and on the balance between pro- and anti-angiogenic factors, especially in RA patients, have not been studied in depth. To the best of our knowledge, only three studies demonstrate that tofacitinib could reduce microvascular angiogenesis [28], reduce pro-angiogenic factors [29], and exert a direct effect on endothelial cell proliferation and migration in vitro and in a mouse model of collagen-induced arthritis (CIA) [30].

In this study, we aimed to investigate the mechanisms that drive the effects of tofacitinib on angiogenesis. We demonstrate, using an in vitro co-culture system of fibroblasts and monocytic cell lines, that tofacitinib enhances endostatin generation by decreasing the phosphorylation of STAT3, leading to increased levels of miR-146a-5p and decreased levels of CD147 and MMP-9, with enhanced activity of secreted proteasome 20S. Moreover, tofacitinib inhibited the degradation of endostatin by cathepsin S, resulting in an extended half-life of endostatin. These findings were also corroborated in serum samples obtained from RA patients that were treated with tofacitinib.

## 2. Results

### 2.1. Tofacitinib Inhibits Angiogenesis in the Co-Culture and Oppositely Regulates MMP-9 and Proteasome 20S Activities

Tofacitinib, a known JAKinib currently in use for the treatment of RA patients, can block the excessive production of pro-inflammatory cytokines. However, its role in inhibiting angiogenesis has not been explored in depth. To explore whether tofacitinib inhibits angiogenesis and by what mechanisms, we employed a human in vitro co-culture system by incubating together a fibrosarcoma cell line (HT1080) and a monocytic cell line (U937). To avoid cell–cell contact and allow extraction of RNA and protein from each cell line, the two cell types were separated by inserts (0.4 μm pore size). As before [13], we added a minimal concentration (1 ng/mL) of the strong MMP-9 inducer TNFα to the culture that did not cause cell death. To explore the effects of tofacitinib, we first determined the concentrations that would not cause cell death but would still affect the pro-angiogenic factors VEGF and MMP-9. These calibration experiments (Figure S1), where HT1080 and U937 cells were co-cultured in the presence of TNFα, allowed us to proceed with a concentration of 1 μM of tofacitinib.

**Figure 1:**
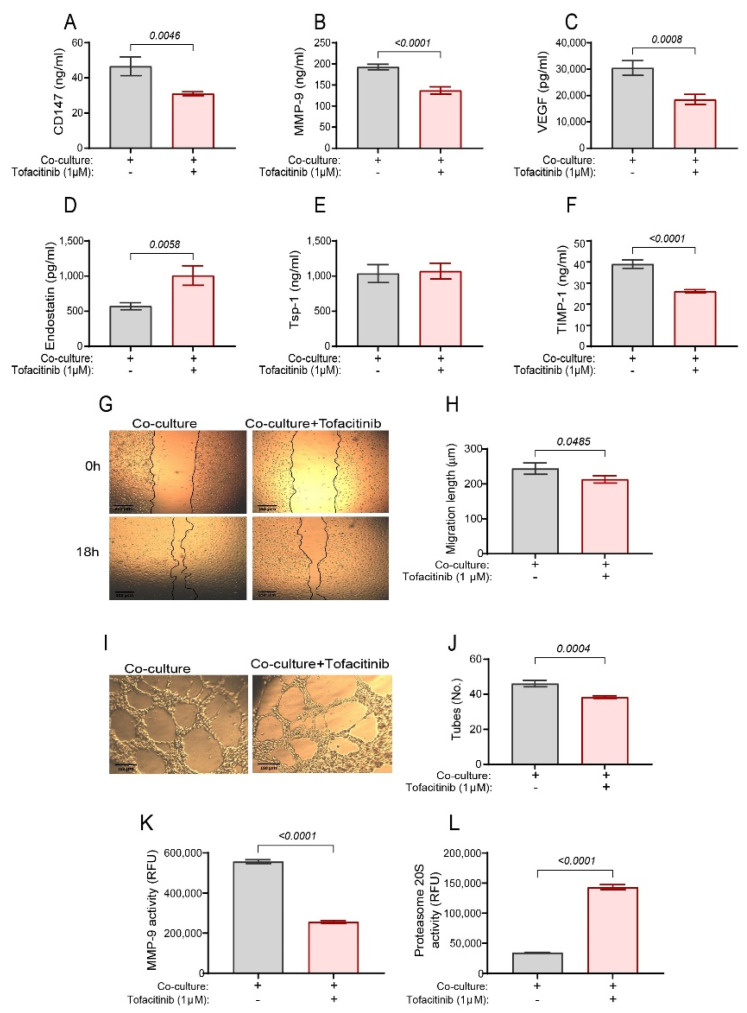
Tofacitinib affects both pro- and anti-angiogenic factors in co-cultured fibroblasts and monocytes. HT1080 fibroblast cells (3 × 10^6^ cells) were co-cultured with U937 cells (at a ratio of 1:1) and with TNFα (1 ng/mL) for 48 h in serum-starvation medium, in the absence or presence of tofacitinib (1 μM). At the end of the incubation, ELISA was used to evaluate the concentrations of (**A**) CD147, (**B**) MMP-9, (**C**) VEGF, (**D**) Endostatin, (**E**) Tsp-1, and (**F**) TIMP-1 (n = 14) in the supernatants. Confluent EaHy926 cells (2 × 10^4^ cells) were scratched and detached cells were washed away. Supernatants derived from the co-cultures (diluted 1:2 with serum-starvation medium) were added to the endothelial layer. (**G**) Representative images at the beginning of the experiment (0 h) and after 18 h (bar size is 250 μM) and (**H**) the migration length calculated as explained in the methods (n = 20). The ability of factors found in the co-cultured supernatants (diluted 1:2) to form tube-like structures was evaluated as explained in the methods. (**I**) Representative images were taken after 18 h of incubation (bar size is 150 μM) and (**J**) the quantification of the number of the closed lumens that were counted (n = 20). Supernatants from co-cultures were evaluated for the activities of (**K**) MMP-9, using the fluorescent substrate DNP-PLGMWSR (50 μM, n = 6), and (**L**) proteasome 20S, using the fluorescent substrate SUC-LLVY-2R110 (15 μM, n = 6). All data are presented as means ± SE and were analyzed using the unpaired two-tailed *t*-test.

When the co-cultured cells were incubated in the presence of tofacitinib (1 μM), the concentrations of the pro-angiogenic factors CD147, VEGF, MMP-9, and TIMP-1 were reduced (by 33%, 39%, 29%, and 33%, respectively, Figure 1A–C,F) and the anti-angiogenic factor Tsp-1 was unchanged (Figure 1E), whereas the anti-angiogenic factor endostatin was increased by 76% (Figure 1D). Additionally, other MMPs that are known to be involved in angiogenesis were not significantly affected by tofacitinib (Figure S2). Thus, the effect of tofacitinib on MMPs is selective and specific to MMP-9.

Furthermore, to examine whether tofacitinib exerts an effect on the overall angiogenic potential, we applied supernatants derived from the co-cultures incubated with or without tofacitinib onto a scratched monolayer of the human endothelial cells EaHy926 or onto these endothelial cells that were grown on a Coultrex^®^ basement membrane to form tube-like structures. Indeed, the presence of tofacitinib inhibited the rate of endothelial cell migration (Figure 1G,H) and reduced the number of closed lumens that represent the ability of the endothelial cells to form tube-like structures (Figure 1I,J).

We have previously demonstrated that endostatin, the product of the proteolytic cleavage of Col18A, is cleaved by the cooperative activities of MMP-9 and proteasome 20S [21]. Therefore, we speculated that tofacitinib could affect one or both of these enzymes and reduce the levels of endostatin levels in the co-cultured cells. Therefore, we next evaluated the activities of MMP-9 and proteasome 20S in the supernatants of co-cultures, with or without tofacitinib. MMP-9 activity was reduced upon the addition of tofacitinib (Figure 1K), whereas proteasome 20S activity was increased (Figure 1L). As the two proteases were shown to cooperate in generating endostatin [21], endostatin levels are predicted to be reduced when the activity of even one of these two enzymes is inhibited. However, although MMP-9 activity was inhibited by tofacitinib, the levels of endostatin were increased. This suggests that other endostatin-generating mechanisms are involved, possibly another protease whose activity may be relatively minor compared to MMP-9 and proteasome 20S that is revealed once tofacitinib disrupts the balance of activities between MMP-9 and proteasome 20S.

### 2.2. The Effects of Tofacitinib on Endostatin Are Partly Mediated by CD147

**Figure 2:**
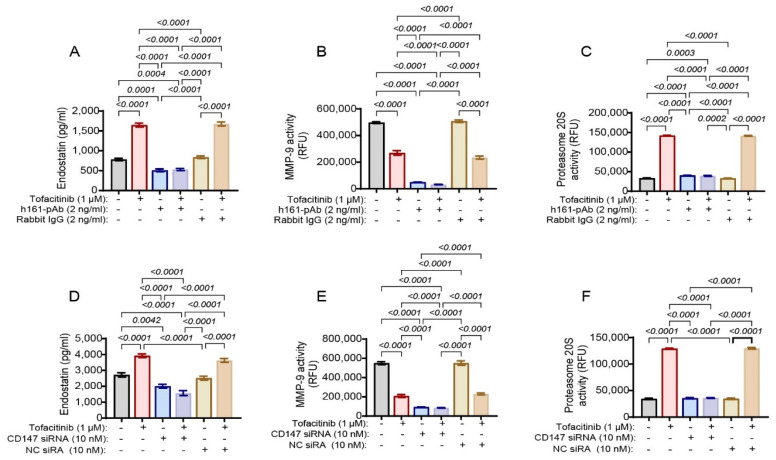
CD147 mediates the activity of tofacitinib. (**A**–**C**) Cells were co-cultured, as described in Figure 1, and either the anti-CD147 antibody (h161-pAb, 2 ng/mL) or irrelevant antibodies (Rabbit IgG, 2 ng/mL) were added to some samples. (**A**) Concentrations of endostatin were determined (n = 9); (**B**) MMP-9 activity (n = 6) and (**C**) proteasome 20S activity (n = 6) were measured. Alternatively, (**D**–**F**) HT1080 cells (10^4^ cells) were first transfected with CD147 siRNA or its negative control NC siRNA (10 nM each) and allowed to recover in full medium. After 24 h of incubation, U937 cells were added at a 1:1 ratio with the addition of TNFα (1 ng/mL) and with or without the addition of tofacitinib (1 μM). (**D**) Concentrations of endostatin (n = 10), (**E**) MMP-9 activity (n = 6), and (**F**) proteasome 20S activity (n = 6) were measured. Data are presented as means ± SE and analyzed using one-way ANOVA followed by Bonferroni’s post hoc test.

To explore whether the effects of tofacitinib on endostatin levels are mediated through CD147, we added the anti-CD147 antibody (h161-pAb) or the irrelevant rabbit IgG alone, or with tofacitinib. Anti-CD147 alone, but not rabbit IgG, decreased endostatin generation (Figure 2A), as well as the activity of MMP-9 (Figure 2B) but not of proteasome 20S (Figure 2C). Moreover, the addition of anti-CD147 in the presence of tofacitinib blocked the increase in endostatin levels (Figure 2A) and in proteasome 20S activity (Figure 2C) observed without the antibody. This phenomenon was repeated when CD147 expression was knocked down (Figure 2D–F). Thus, CD147 expression and activity are necessary for the manifestation of the effects of tofacitinib, suggesting that CD147 is downstream of the signaling initiated by tofacitinib.

### 2.3. Tofacitinib Inhibits CD147 Expression through STAT3 and miR-146a-5p

**Figure 3:**
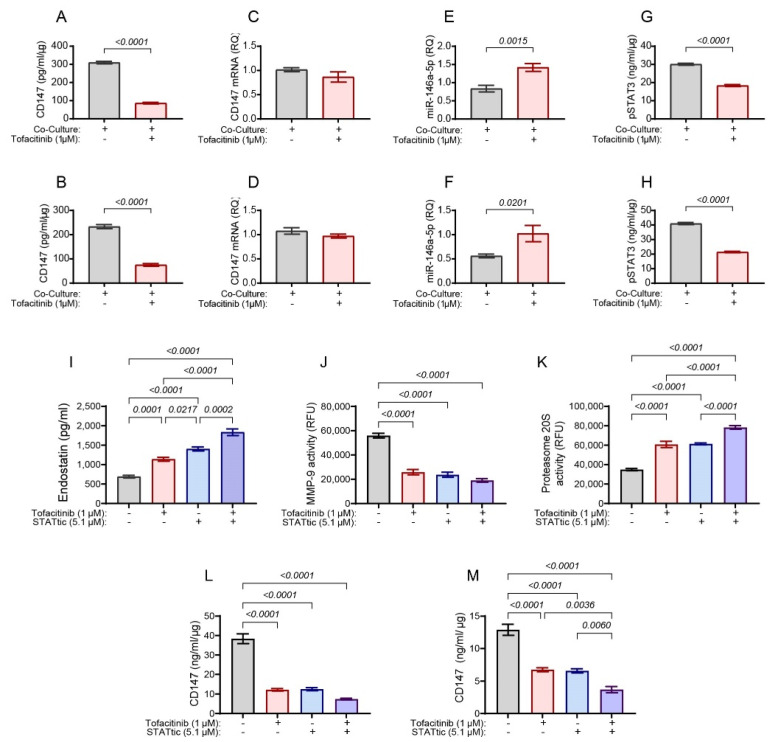
Tofacitinib inhibits CD147 expression through STAT3 and miR-146a-5p. Cells were co-cultured as described in Figure 1. The co-culture was treated with or without tofacitinib (1 μM) (Panels (**A**–**H**)); or with tofacitinib, the STAT3 inhibitor STATtic (5.1 μM); or their combination (panels (**I**–**M**)). Supernatants were collected and cells were lysed to extract total protein or RNA. (**A**,**C**,**E**,**G**) HT1080 cell lysates. (**B**,**D**,**F**,**H**) U937 cell lysates. (**A**,**B**) The concentrations of CD147 protein in cell lysates were determined by ELISA (n = 6). (**C**,**D**) CD147 mRNA was determined by qPCR (n = 5). (**E**,**F**) The levels of miR-146a-5p were determined by qPCR (n = 7). (**G**,**H**) The levels of phosphorylated STAT3 were determined by ELISA (n = 6). Levels of CD147 mRNA and miR-146-5p were determined relative to their expression in HT1080 incubated alone in the absence of tofacitinib. Data are presented as means ± SE and analyzed using the unpaired two-tail student *t*-test. The concentrations of (**I**) secreted endostatin (n = 6), (**J**) MMP-9 activity (n = 6), and (**K**) proteasome 20S activity (n = 6) were measured in the supernatants. The expression of CD147 protein in (**L**) HT1080 cell lysates and (**M**) in U937 cell lysates was evaluated by ELISA (n = 6). Data are presented as means ± SE and analyzed using one-way ANOVA followed by Bonferroni’s post hoc test.

Because the effects of tofacitinib on endostatin levels are partially mediated through CD147, we asked whether tofacitinib directly affects CD147 expression or activity. To this end, we first examined CD147 protein expression levels in the lysates obtained from the co-cultured fibroblasts and monocytic cells that were separated by inserts. Tofacitinib markedly reduced CD147 protein levels in both the HT1080 and U937 cells when they were co-cultured (Figure 3A,B); however, CD147 mRNA expression was not changed (Figure 3C,D). This indicates that the regulation of CD147 is post-transcriptional. We also found that tofacitinib enhanced miR-146a-5p levels in both HT1080 and U937 cells (Figure 3E,F). As we previously demonstrated that CD147 is regulated by miR-146a-5p [13,31], we conclude that the reverse correlation between miR-146a-5p levels and CD147 protein levels further indicates that miR-146a-5p regulates CD147 expression. Lastly, tofacitinib reduced the phosphorylation of STAT3 in both HT1080 and U937 cells when co-cultured (Figure 3G,H).

To prove that STAT3 regulates the levels of CD147 protein, we incubated the cells with the specific STAT3 inhibitor STATtic (5.1 μM), with or without tofacitinib (1 μM). The inhibition of STAT3 with STATtic increased endostatin levels (Figure 3I), as well as the activity of proteasome 20S (Figure 3K), and decreased the activity of MMP-9 (Figure 3J). Tofacitinib, when added alone, exhibited similar effects. The addition of STATtic together with tofacitinib enhanced endostatin and proteasome 20S activity even further but did not change the activity of MMP-9 relative to either agent alone. Additionally, inhibition of STAT3, tofacitinib, or their combination decreased CD147 protein levels in lysates of both HT1080 and U937 cells that were co-cultured (Figure 3L,M). Thus, tofacitinib (as a JAKinib) has similar effects to the STAT3 inhibitor and STAT3 is involved in the regulation of CD147. However, the added effect of the combined addition of STATtic and tofacitinib relative to either of them alone may suggest that tofacitinib influences endostatin and proteasome 20S not only through its inhibitory effect on STAT3 but possibly through other STATs as well.

### 2.4. Tofacitinib Reveals the Activity of Another Protease That Cleaves Endostatin

**Figure 4:**
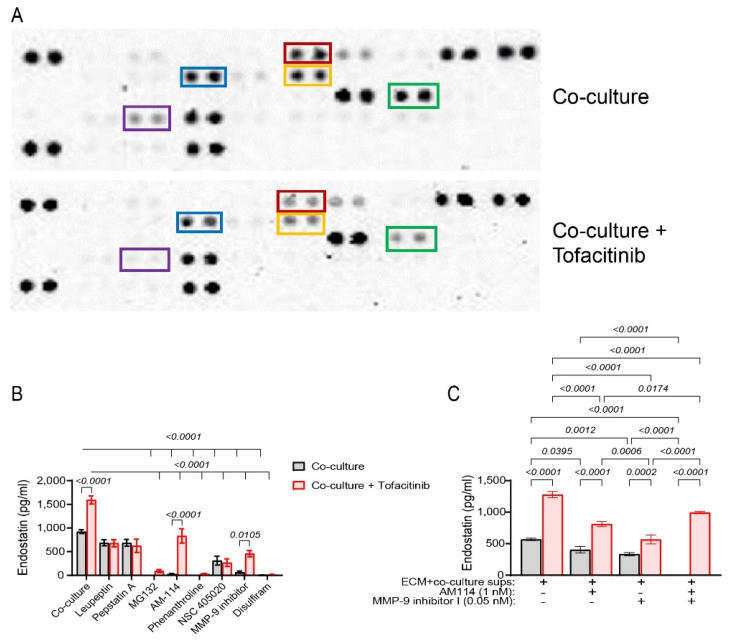
Cathepsin S may participate in the generation of endostatin and tofacitinib reverses the inhibition of MMP-9 and proteasome 20S. (**A**) Supernatants derived from co-cultures or co-cultures incubated with tofacitinib were applied onto the membranes of the Proteome Profiler Protease Human Protease Array kit. Tofacitinib inhibited the secretion of cathepsin A (red box), cathepsin S (blue box), cathepsin X/Z/P (yellow box), MMP-2 (green box), and MMP-8 (purple box). However, only cathepsin S has been previously associated with the generation of endostatin. (**B**) The HT1080 cells were co-cultured with the U937 cells (3 × 10^4^ cells each) in serum-starvation medium and incubated for 48 h in the presence of TNFα (1 ng/mL), with or without the addition of tofacitinib (1 μM) and the different protease inhibitors (Table S1). Endostatin levels were evaluated by ELISA (n = 10). Data are presented as means ± SE and analyzed using two-way ANOVA followed by Dunnett’s post hoc test. (**C**) HT1080 (3 × 10^4^ cells) were incubated in a serum-starvation medium with TNFα (1 ng/mL) for 72 h and allowed to deposit their ECM proteins. Cells were destroyed with double distilled water (DDW) for 20 min and the cellular debris was washed away with PBS three times. The remaining ECM proteins were incubated with supernatants derived from the co-cultures with or without tofacitinib treatment (1 μM), and with indicated limiting concentrations of the inhibitors AM114, MMP-9 inhibitor I, or their combination. Endostatin levels were evaluated by ELISA (n = 6). Data are presented as means ± SE and analyzed using two-way ANOVA followed by Bonferroni’s post hoc test.

To identify which protease mediates the increased levels of endostatin during tofacitinib inhibition, the Proteome Profiler Protease Array kit was used. Tofacitinib inhibited the secretion of several cathepsins (cathepsin A, S, X/Z/P), as well as that of MMP-2 and MMP-8 (Figure 4A). However, these proteases, with the exception of cathepsin S, are not known to cleave Col18A and might, therefore, be irrelevant.

We also used several general and specific inhibitors of proteases that were added to the co-culture, with or without the presence of tofacitinib (their concentrations are listed in Table S1). Tofacitinib promoted the secretion of endostatin in the uninhibited co-cultured cells, as seen before; however, the addition of pepstatin A and leupeptin, which inhibit different cathepsins, prevented this elevation in endostatin levels (Figure 4B). As expected, the general and specific inhibitors of MMP-9 and proteasome 20S abolished the accumulation of endostatin; however, tofacitinib reversed this inhibition, although not to the levels measured in the absence of the inhibitors.

We have previously shown that secreted MMP-9 and proteasome 20S cooperate outside the cells to generate endostatin. This was demonstrated in the non-cellular system that contained only ECM proteins deposited by HT1080 cells that were incubated for 72 h and then removed by double distilled water, to which we added supernatants obtained from treated cells [21]. We have used this non-cellular system, with the addition of the AM114 and MMP-9 inhibitor I in limited concentrations, together with supernatants derived from the co-cultured cells incubated with or without tofacitinib. AM114 and MMP-9 inhibitor I both inhibited endostatin accumulation and their combination completely abrogated it (Figure 4C). Tofacitinib enhanced the level of endostatin in non-treated supernatants as well as in the presence of the AM114 and MMP-9 inhibitor I, alone or in combination; although, in the presence of the inhibitors, tofacitinib did not fully restore the levels of endostatin shown in their absence.

These results suggest that tofacitinib affects the accumulation of endostatin in a pathway that is different from the synergistic activity of proteasome 20S and MMP-9. Such a pathway may involve a protease that partially compensates for the absence of the activities of either MMP-9 or proteasome 20S. Alternatively, it could involve a protease that affects the steady-state levels of the endostatin protein. The obvious candidate for such a protease, based on the results of the Proteome Profiler Array and the inhibition by pepstatin A and leupeptin, is cathepsin S. We note that the previous results showing that tofacitinib also affects MMP-9 activity and proteasome 20S activity are not mutually exclusive with the conclusion that another protease is involved.

### 2.5. Tofacitinib Does Not Affect Col18A Transcription or Deposition but Inhibits the Degradation of Endostatin in Co-Cultures

**Figure 5:**
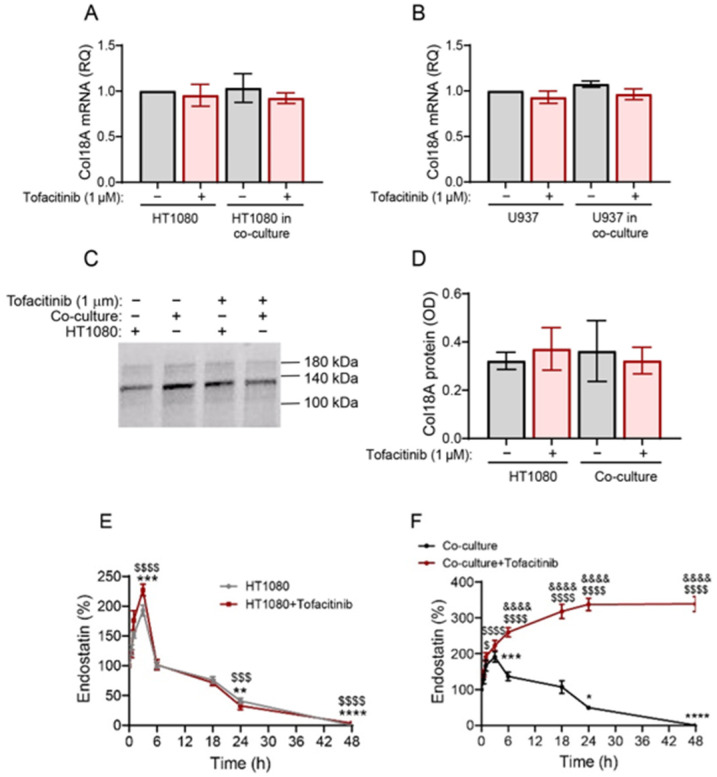
Tofacitinib does not affect Col18A synthesis but prevents endostatin degradation in the co-cultures. The human HT1080 cell or the U937 cells (3 × 10^6^ cells each) were seeded as single cultures or in co-cultures in serum-starvation medium and incubated for 48 h in the presence of TNFα (1 ng/mL), with or without tofacitinib (1 μM). The U937 cells were co-cultured in inserts (0.4 μm pore size) to allow separate extraction of protein or RNA from each cell type. After 48 h, supernatants were collected and total RNA was extracted, transcribed to cDNA, and amplified by qPCR. (**A**) The accumulation of Col18A mRNA in HT1080 cells cultured in single- and co-cultures (n = 8). (**B**) The accumulation of Col18A mRNA in U937 cells incubated in single- and co-cultures (n = 8). The means ± SE are presented and changes are analyzed using one-way ANOVA followed by Bonferroni’s post hoc test. Total protein concentrations in the deposited ECM were determined by Branford reagent and 15 μg of total protein were uploaded in each lane. (**C**) A representative image of a Western Blot demonstrating Col18A protein levels and (**D**) its quantitation (n = 3). (**E**,**F**) Degradation of endostatin in the non-cellular system: HT1080 cells (3 × 10^4^ cells) were incubated in serum-starvation medium with TNFα (1 ng/mL) for 72 h and allowed to deposit their ECM proteins and then incubated for 20 min with DDW. The de-cellularized ECM was washed with PBS three times to remove cellular debris. Supernatants derived from the tofacitinib-treated cells in single- or co-cultures were added to the ECM for 24 h to generate endostatin. Then, the MMP-9 inhibitor I (0.15 nM) and the proteasome 20S inhibitor AM114 (3 nM) were added to prevent the generation of new endostatin and the levels of endostatin were determined in the samples at different time points (n = 6). Data are presented as means ± SE and analyzed using two-way ANOVA followed by Bonferroni’s post hoc test. *, *p* < 0.05, **, p<0.01, ***, *p* < 0.001, ****, *p* < 0.0001 relative to HT1080 or co-culture at time 0 h; $, *p* < 0.05, $$$, *p* < 0.001, $$$$, *p* < 0.0001, relative to HT1080 with tofacitinib or co-culture with tofacitinib at time 0 h; &&&&, *p* < 0.0001 relative to the co-culture at the same time point.

To explore the possibility that tofacitinib regulates the steady-state levels of endostatin, we first asked whether the inhibition of the JAK–STAT pathway by tofacitinib affects the transcription of Col18A. We found that tofacitinib does not change the steady-state levels of Col18A mRNA in either HT1080 or U937 cells when they are co-cultured (Figure 5A,B). The deposition of Col18A in the ECM is also unchanged, as demonstrated by the WB analysis (Figure 5C,D). Therefore, tofacitinib has no effect on the synthesis of the endostatin precursor Col18A.

To investigate whether tofacitinib affects the degradation of endostatin, we used the non-cellular system and added supernatants derived from the different treatments to the ECM for 24 h. This allowed the MMP-9 and proteasome 20S activities to generate endostatin. After 24 h, when endostatin was already generated in the system, we added the MMP-9 inhibitor I and the proteasome 20S inhibitor AM114 to stop the generation of new endostatin molecules. This approach allowed us to determine endostatin concentrations over time. We show that the rate of endostatin degradation remains unchanged by tofacitinib in the HT1080 single cultures, with an approximate half-life of 24 h (Figure 5E). However, when tofacitinib was added to cells incubated in co-culture, it completely inhibited endostatin degradation (Figure 5F).

These results suggest that the degradation of endostatin, after the first 24 h, in HT1080 single culture (with or without tofacitinib) and in co-culture without tofacitinib, is not carried out by either MMP-9 or proteasome 20S, as these were inhibited. Therefore, their role is limited only to the generation of new endostatin cleaved from its precursor Col18A. This is also supported by the previous result showing an accumulation of endostatin when the AM114 and MMP-9 inhibitor I were administered (Figure 4C). Moreover, these results suggest that another protease that is contributed to by the U937 cells and is inhibited by tofacitinib is degrading endostatin. Based on our previous results that show a reduction in cathepsin S in the protease profiler array (Figure 4A), we predicted that cathepsin S is that protease, although other MMPs or cathepsins, such as cathepsin A or X/Z/P, cannot be ruled out at this point.

### 2.6. Cathepsin S Degrades Endostatin and Tofacitinib Inhibits This Degradation and Extends the Half-Life of Endostatin

**Figure 6:**
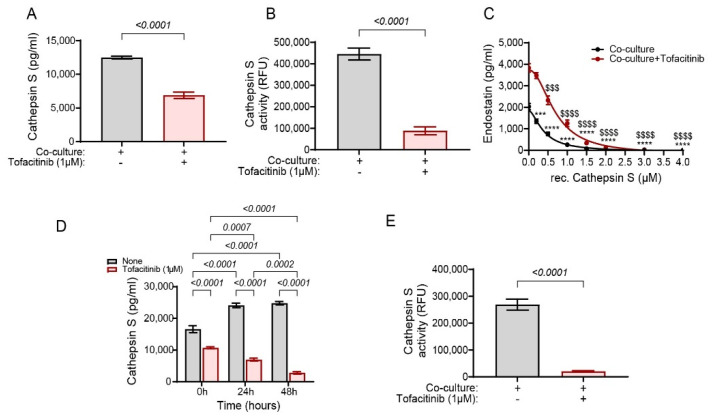
Tofacitinib inhibits cathepsin S and allows the accumulation of endostatin. (**A**) The concentration of cathepsin S and (**B**) its activity were evaluated in the supernatants derived from the co-culture experiment (n = 6). (**C**) The generation of endostatin was measured in the non-cellular system. Supernatants derived from the degradation experiment (as explained in Figure 5F), that were obtained from co-cultured cells with or without the addition of tofacitinib (1 μM) were added to the ECM system for 24 h incubation to generate the maximal amount of endostatin. Then the MMP-9 inhibitor I (0.15 nM) and AM114 (3 nM) were added to prevent the generation of new endostatin, together with the addition of increasing concentrations of rec. cathepsin S, and the levels of endostatin were determined (n = 6 in each concentration). (**D**) The concentrations of cathepsin S in the degradation experiment were evaluated in different periods and (**E**) cathepsin S activity was assessed in the degradation experiment at 48 h (n = 6). Data are presented as means ± SE and analyzed using (**A**,**B**,**E**) an unpaired, two-tailed student *t*-test, (**C**) two-way ANOVA followed by Bonferroni’s post hoc test, and (**D**) one-way ANOVA followed by Bonferroni’s post hoc test. ***, *p* < 0.001, ****, *p* < 0.0001 relative to co-culture at time 0 h; $$$, *p* < 0.001, $$$$, *p* < 0.0001, relative to co-culture with tofacitinib at time 0 h.

To test our hypothesis that cathepsin S is responsible for the degradation of endostatin and is inhibited by tofacitinib, we first measured its levels in the supernatants of the co-cultures. Both the concentrations of cathepsin S and its activity demonstrated a significant reduction in the presence of tofacitinib (Figure 6A,B). Furthermore, when recombinant cathepsin S was added to the supernatants derived from the co-culture with and without tofacitinib, and in the presence of the protease inhibitors that prevented the generation of new endostatin (as was previously described for the degradation experiment), it inhibited the accumulation of endostatin previously observed in the co-culture with tofacitinib (Figure 6C). The reduction in cathepsin S concentrations in the presence of tofacitinib was time-dependent (Figure 6D) and at 48 h tofacitinib blocked the activity of cathepsin S (Figure 6E). Thus, tofacitinib inhibited the secretion and activity of cathepsin S, which resulted in the accumulation of endostatin, and this effect could be reversed by adding recombinant cathepsin S.

### 2.7. Tofacitinib Reduces Angiogenesis in RA Patients

To verify that our in vitro findings correlate to the clinical effects in a cohort of tofacitinib-treated RA patients, we collected serum samples from RA patients before the beginning of the treatment (V1) and then after one month (V2), three months (V3), and six months (V4). Only 14 patients out of the 20 RA patients received the full treatment for 6 months and, therefore, the cohort is very small. The demographic and clinical data of these patients, who were classified according to the DAS28 score as responders to treatment if the DAS28 difference (delta DAS28) was more/equal to 1.2, and as non-responders if the difference was less than 1.2 [32], are summarized in Table 1.

**Figure 7:**
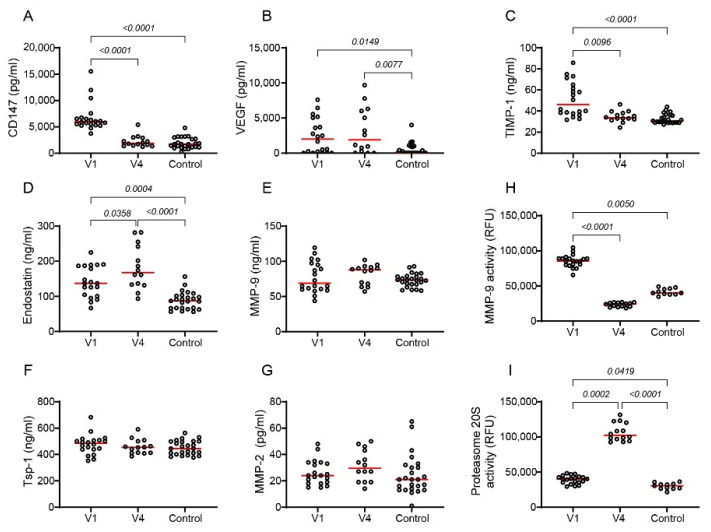
Tofacitinib affects both pro- and anti-angiogenic factors in serum samples of RA patients. (**A**–**G**) The concentrations of pro- and anti-angiogenic factors were determined in serum samples collected from patients with active RA before the beginning of treatment (V1, n = 20) and after six months of treatment with tofacitinib (V4, n = 14) and in the serum of healthy volunteers (n = 25). (**H**,**I**) The proteasome 20S and MMP-9 activities were assessed in these serum samples. Median values (red bar) are presented and data were analyzed using the non-parametric Kruksal–Wallis ANOVA test followed by Dunn’s multiple comparisons test.

The effects of tofacitinib on the pro-angiogenic and anti-angiogenic factors were measured before and 6 months after the onset of treatment with tofacitinib (Figure 7). The data on the interim period (V2 and V3) are provided in Figure S3 and show the gradual and accumulating effect of tofacitinib. Tofacitinib reduced the levels of CD147 and TIMP-1, resulting in levels similar to those of the controls after 6 months (Figure 7A,C). In contrast, endostatin levels were elevated even before treatment began relative to the controls and tofacitinib further elevated its levels (Figure 7D). Tofacitinib did not change the serum levels of VEGF, MMP-9, MMP-2, and Tsp-1, (Figure 7B,E–G) suggesting a selective impact. However, despite the lack of change in its serum level, tofacitinib reduced the activity of MMP-9 (Figure 7H). In contrast, tofacitinib enhanced the activity of proteasome 20S (Figure 7I). Additionally, looking at the data from V1 and V4, serum levels of CD147 were correlated with MMP-9 activity, proteasome 20S activity, and TIMP-1, but not with endostatin, and were correlated with several parameters of disease severity (Table 2).

**Figure 8:**
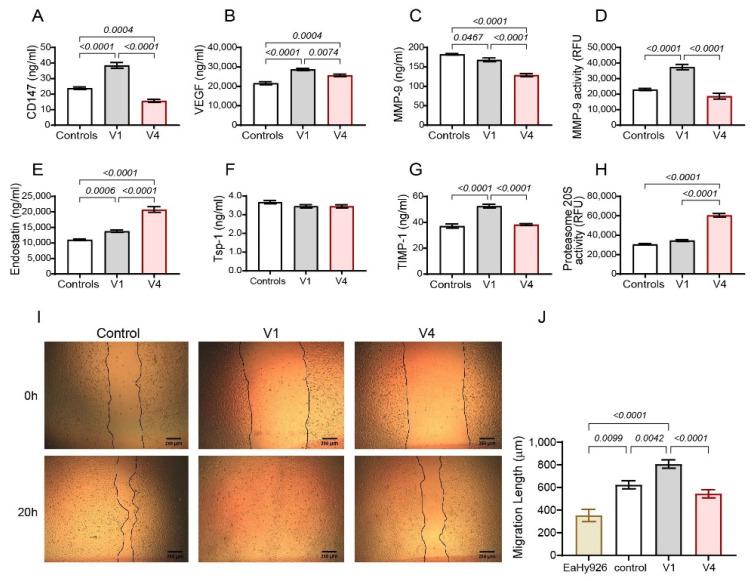
Serum from tofacitinib-treated RA patients reduces pro-angiogenic factors and enhances endostatin generation in HT1080 fibroblast cells. Serum samples (diluted 1:2) obtained from the study groups were added onto HT1080 cells (3 × 10^4^) that were incubated in serum-starvation medium with TNFα (1 ng/mL). Concentrations of (**A**) CD147, (**B**) VEGF, (**C**) MMP-9, (**E**) endostatin, (**F**) Tsp-1, and (**G**) TIMP-1 were determined by ELISA after 48 h of incubation (n = 13). (**D**) MMP-9 and (**H**) proteasome 20S activities were assessed (n = 10–13 in each group). The wound assay (described in detail in the methods) was employed using serum samples from the study groups (diluted 1:2). (**I**) Representative and (**J**) the migration distance (n = 10–12 in each group). Data are presented as means ± SE and analyzed using the one-way ANOVA test followed by Bonferroni’s post hoc test.

To corroborate the in vitro findings further, we examined the effect of serum samples obtained from RA patients, before treatment and after 6 months of tofacitinib treatment, for their ability to induce endostatin generation and affect MMP-9 and proteasome 20S activities when added to cultured HT1080 cells. In accordance with the in vitro findings, HT1080 cells that were incubated with serum samples from RA patients before treatment (V1) increased the levels of secreted CD147 relative to the controls and tofacitinib reduced them (Figure 8A). The levels of endostatin in the supernatants of HT1080 cells incubated with V1 samples were increased relative to the controls and the treatment with tofacitinib enhanced these levels even further (Figure 8E). Again, in agreement with the in vitro findings, the levels of TIMP-1 expression (Figure 8G), MMP-9 expression (Figure 8C), and MMP-9 activity (Figure 8D) were decreased after 6 months of tofacitinib treatment, whereas the levels of proteasome 20S were enhanced (Figure 8H).

The angiogenic potential of the serum samples was assessed by the wound assay. While serum samples from healthy individuals showed enhanced migration relative to the basal migration of the endothelial cells, reflecting the effect of growth factors in the human serum, the samples from untreated patients enhanced it even further. However, 6 months of treatment with tofacitinib reduced this potential to levels below those of the controls (Figure 8I,J).

**Figure 9:**
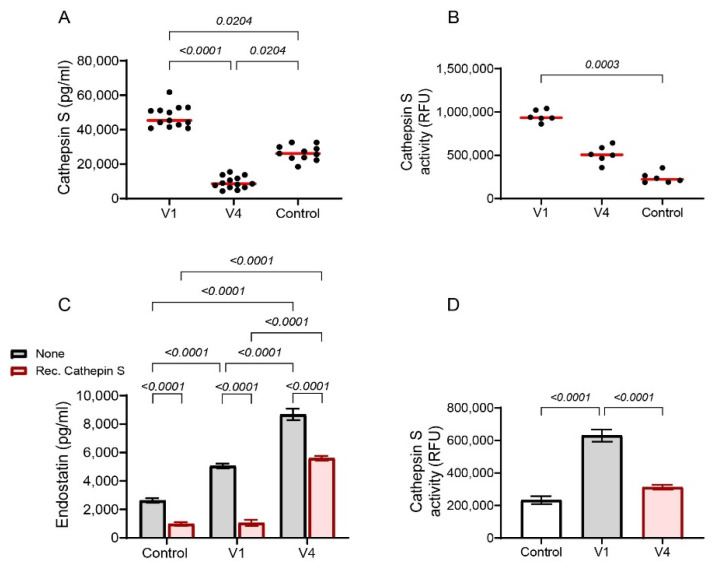
Cathepsin S is decreased in tofacitinib-treated RA patients. (**A**) Levels of cathepsin S (n = 13) and (**B**) the activity of cathepsin S (n = 6) were measured in the serum samples obtained from RA patients before treatment (V1) and after 6 months of treatment with tofacitinib (V4). Median values are presented (red line) and differences were analyzed using the non-parametric Kruksal–Wallis ANOVA followed by Dunn’s multiple comparisons test. (**C**) Serum samples from RA patients or controls were diluted 1:2 and added to the HT1080 and U937 co-cultures, with or without the addition of recombinant cathepsin S (1 μM) (n = 13). (**D**) The activity of cathepsin S in serum samples that were added to the co-cultured cells without the addition of recombinant cathepsin S (n = 6). Data are presented as means ±SE and were analyzed using one-way ANOVA followed by Bonferroni’s post hoc test.

Finally, to corroborate the role of cathepsin S in the degradation of endostatin in vivo, we measured it in serum samples from RA patients and from the healthy controls and observed a reduction in cathepsin S concentrations and activity after 6 months of tofacitinib treatment relative to the samples before treatment (Figure 9A,B). The addition of diluted serum samples obtained from patients after 6 months of tofacitinib treatment to co-cultured cells resulted in increased levels of endostatin (Figure 9C) and the activity of cathepsin S was reduced accordingly (Figure 9D). In contrast, the addition of recombinant cathepsin S to the same co-culture reduced the levels of endostatin (Figure 9C). Thus, tofacitinib reduces the levels of cathepsin S and its activity, resulting in increased endostatin levels in RA patients.

## 3. Discussion

Tofacitinib is known to be an effective inhibitor of the JAK–STAT pathway and is used to inhibit the production of many pro-inflammatory cytokines that are enhanced in RA patients [24,33,34]. Here, we show that in addition to its anti-inflammatory properties, tofacitinib inhibits angiogenesis by reducing the pro-angiogenic factors and elevating the levels of the anti-angiogenic factor endostatin in RA patients.

We show that tofacitinib reduces the levels of the pro-angiogenic factors CD147 and TIMP-1 both in an in vitro co-culture system of fibroblast and monocytic cell lines (Figure 1) and in the serum samples obtained from RA patients treated with tofacitinib (Figure 7). The levels of the pro-angiogenic MMP-9 were reduced in the co-culture but not in the serum samples, but MMP-9 activity was reduced in both. In contrast, tofacitinib increased the levels of endostatin in both systems. Indirectly, serum samples from tofacitinib-treated RA patients could reduce the levels of CD147, MMP-9, VEGF, and TIMP-1 secreted from HT1080 fibroblasts and enhance the levels of endostatin (Figure 8). Moreover, the angiogenic potential that was manifested by the wound assay and the tube formation assay also demonstrated an inhibitory effect of tofacitinib on angiogenesis (Figure 1 and Figure 8). The effects of tofacitinib were selective, as it did not affect the expression levels of Tsp-1, MMP-2, MMP-7, or MMP-14, which are also considered potent angiogenic factors. Therefore, we conclude that tofacitinib inhibits angiogenesis by shifting the balance between pro-angiogenic and anti-angiogenic factors in favor of the latter.

CD147 is considered a pro-angiogenic factor, as it induces the expression of VEGF and MMP-9, even in its secreted form [14,35]. CD147 also enhances TIMP-1 [36], the selective inhibitor of MMP-9, which is generally considered an anti-angiogenic factor, but can also have pro-angiogenic activities [37,38]. Moreover, in a recent study, we have shown that CD147 regulates the activities of secreted MMP-9 and proteasome 20S that cleave col18A to produce endostatin [21]. Collectively, these data place CD147 as a central regulator of angiogenesis. Therefore, we asked whether the inhibitory effect of tofacitinib is a result of its specific effect on CD147. By adding tofacitinib to cells where CD147 was neutralized with the anti-CD147 antibody h161-pAb or its expression was silenced, we observed that the tofacitinib-mediated increase in endostatin levels and proteasome 20S activity were blocked (Figure 2). Most importantly, the correlation found between CD147 and the different parameters that make up the severity of the arthritic disease (Table 2) confirms the central role of CD147 in mediating the effects of tofacitinib. Thus, the presence of CD147 is important in mediating at least some of the effects of tofacitinib on angiogenesis. Although we show that CD147 mediates the effects of tofacitinib on the levels of endostatin, these two proteins show no correlation, suggesting the involvement of another factor. Such a factor is likely cathepsin S, which degrades endostatin and is inhibited by tofacitinib. Thus, endostatin levels depend on both CD147 and cathepsin S and, therefore, were not correlated to CD147.

The finding that the effects of tofacitinib are mediated through CD147 raises the question of how tofacitinib affects CD147. The regulation of CD147 expression is hardly understood. CD147 is known to be regulated at post-transcriptional levels and we and others have shown that miR-146a-5p is involved in its regulation [13,31,39]. However, the JAK–STAT pathway that tofacitinib inhibits was implicated in the regulation of CD147 expression only in one study, which suggested that STAT1, but not STAT3, is involved in the regulation of CD147 expression [40]. As STAT3 is involved in angiogenesis [27], and tofacitinib affects its phosphorylation [41], we decided to look at the effects of tofacitinib on STAT3 in our in vitro co-culture system.

**Figure 10:**
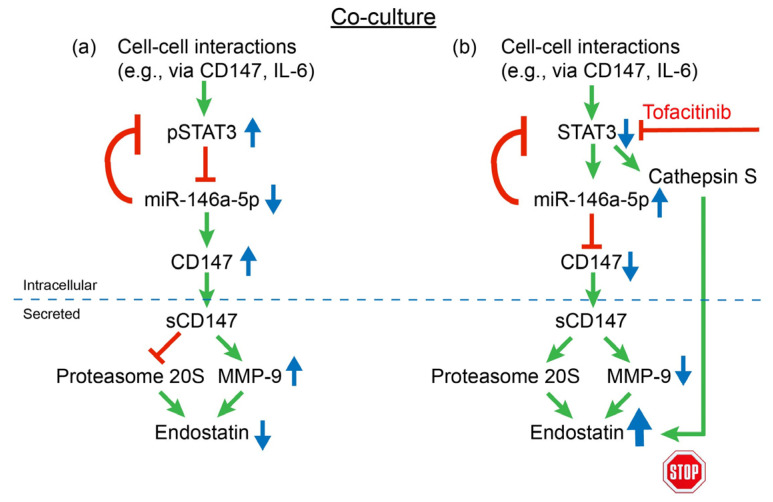
A schematic model of the effects of tofacitinib on endostatin. Tofacitinib works by two mechanisms: (**a**) Tofacitinib inhibits STAT3 phosphorylation, allowing the increase in miR-146a-5p levels that, in turn, reduce CD147 protein levels and CD147 regulates MMP-9 and proteasome 20S activities; (**b**) Tofacitinib inhibits the secretion and activity of cathepsin S that degrades endostatin and, thus, tofacitinib inhibits angiogenesis by affecting both pro-angiogenic and anti-angiogenic factors. Green arrows suggest activation; red blocked arrows suggest inhibition; Blue arrows indicate the expression of the protein.

We show that relative to the untreated co-culture, tofacitinib reduced CD147 levels, but not its mRNA, in both HT1080 and U937 cells, indicating that tofacitinib regulates CD147 post-transcriptionally. Tofacitinib also increased the expression of miR-146a-5p and reduced the phosphorylation of STAT3 (Figure 3). STAT3 and miR-146a-5p have been linked in other systems as well. STAT3 has been shown to regulate the expression of miR-146a-5p in hepatocarcinoma cells [42] and overexpression of miR-146a-5p was shown to reduce IL-6 levels, STAT3 phosphorylation, and VEGF production in human retinal endothelial cells (RECs) [43]. The lncRNA XIST was shown to act as a sponge for miR-146a-5p and reduce its activity, thereby releasing STAT3 from its inhibitory effect. In contrast, STAT3 enhanced the transcription of lncRNA XIST, establishing a positive feedback loop in pulmonary microvascular endothelial cells [44]. We therefore suggest that in our system, such a loop may also exist, with CD147 being one of its targets (Figure 10). While these results demonstrate the involvement of STAT3 in mediating the effects of tofacitinib, other STAT proteins could also be influenced by this inhibitor. We therefore used the specific STAT3 inhibitor STATtic in combination with tofacitinib. We observed that the combination increased the levels of endostatin and proteasome 20S activity more than each inhibitor alone, suggesting the possible involvement of STAT1, STAT5, or STAT6 in these effects. However, we did not follow up on this further.

In a previous study, we have shown that MMP-9 and proteasome 20S cooperate outside the cells to cleave col18A and generate endostatin and, when one of these two enzymes is inhibited, the production of endostatin is reduced [21]. However, although MMP-9 was inhibited by tofacitinib (Figure 1), the accumulation of endostatin was increased, rather than abolished. Moreover, the use of the MMP-9 inhibitor I or the proteasome 20S specific inhibitor AM114 in the co-culture system (Figure 4B) or in the non-cellular ECM system (Figure 4C), demonstrated that endostatin levels were reduced, but the addition of tofacitinib in their presence increased endostatin levels. These results indicate the involvement of another protease in the production of endostatin.

To identify this protease, we used the Proteome Profiler Protease Array (Figure 4A), which suggested cathepsin S as the most probable candidate. The ability of the cathepsin inhibitors leupeptin and pepstatin A to prevent the tofacitinib-induced increase in endostatin levels also suggested that a cathepsin, probably cathepsin S, is the culprit. Cathepsin S has been implicated before in the breakdown of endostatin [19]. To show the relevance of cathepsin S in our co-culture system, we first demonstrated that tofacitinib does not affect the synthesis of col18A, the precursor of endostatin, at the mRNA or protein levels (Figure 5A–D). Then, we demonstrated that the degradation of endostatin was unaffected by tofacitinib in a single HT1080 culture but, when HT1080 cells were co-cultured with U937 monocytes, the steady-state levels of endostatin were increased in the presence of tofacitinib (Figure 5E,F). Since we blocked the production of new endostatin molecules by inhibiting both MMP-9 and proteasome 20S activities, this suggests that the degradation of endostatin was prevented by tofacitinib, allowing endostatin to accumulate freely in the supernatants, and that the origin of the inhibited protease was monocytic. While we did not rule out the involvement of other proteases in the degradation of endostatin, we have shown that in tofacitinib-treated co-cultures, cathepsin S is the likely protease to mediate endostatin degradation, as tofacitinib inhibited the expression and activity of cathepsin S in the co-cultures over time and increasing amounts of recombinant cathepsin S could degrade endostatin (Figure 6). These in vitro results were strengthened by showing that tofacitinib reduced the levels and activity of cathepsin S in the serum samples of the treated patients and that the addition of recombinant cathepsin S to diluted serum samples that were incubated with co-cultured cells inhibited the tofacitinib-mediated accumulation of endostatin (Figure 9). As cathepsin S can be regulated by STAT3 [45], tofacitinib is likely to inhibit its transcription; however, we did not check this point.

Based on the results presented here, we suggest that tofacitinib, in addition to its inhibitory effects on inflammation and cytokine production, can affect angiogenesis in two major mechanisms. First, tofacitinib, as an inhibitor of JAK–STAT, inhibits STAT3 phosphorylation, which causes an increase in miR-146a-5p levels. As this microRNA directly regulates CD147 expression, its elevated levels reduce CD147 expression, thus decreasing MMP-9 activity and enhancing proteasome 20S activity, as we have shown previously [21]. The second mechanism is that tofacitinib inhibits the secretion and activity of cathepsin S and, thereby, inhibits the degradation of endostatin. Thus, tofacitinib enhances the generation of endostatin from Col18A and reduces its degradation, overall raising its levels (Figure 10) and, thereby, attenuating angiogenesis.

## 4. Materials and Methods

### 4.1. Cultured Cells

We cultured the human fibrosarcoma cell line HT1080 (ATCC CCL-12012) and the monocyte-like cells U937 (ATCC CRL-1593), as described in detail in [21]. Briefly, HT1080 cells were cultured in DMEM with 10% FCS, 1% L-glutamine, 1% amphotericin B, 1% non-essential amino acids (NEAA), and 1% antibiotics, supplemented with 1:3 diluted conditioned media from the HL-60 cells (ATCC CRL-240) that contains secreted FGF-2. The U937 cells were incubated in RPMI-1640 medium with 10% FCS, 1% L-glutamine, 1% amphotericin B, and 1% antibiotics. The human endothelial cell line EaHy926 (ATCC CRL-2922) was cultured in DMEM with 10% FCS, 1% glutamine, 2% HAT (hypoxanthine, aminopterin, thymidine), 1% amphotericin B, and 1% antibiotics. All reagents were from Biological Industries, Beit Ha’emek, Israel. Cells were routinely split twice a week at a 1:3–1:5 ratio and regularly tested for the presence of mycoplasma.

HT1080 (3 × 10^5^ cells/800 μL or 3 × 10^4^ cells/100 μL) were seeded in 24- or 96-well plates and incubated overnight to allow their adherence to the plate. The medium was replaced with a serum-starvation medium (HT1080 medium without FCS supplemented with 0.1% BSA) to avoid the masking of signals. U937 monocytes were added to the co-culture at a ratio of 1:1 and were separated by inserts (0.4 μm pore size) to allow extraction of RNA and proteins from each cell type and prevent their contact. The co-cultured cells were incubated for 48 h with the strong MMP-9 inducer TNFα (1 ng/mL, R&D systems, Minneapolis, MN, USA) and with or without tofacitinib (1 μM, Pfizer, New York, NY, USA). Some experiments included the addition of different protease inhibitors (listed in Table S1), with the STAT3 inhibitor STATtic (5 μM, R&D systems,) or with the rabbit anti-human CD147 antibody (h161-pAb, 2 ng/mL, produced in our lab). In other experiments, the human recombinant cathepsin S (R&D systems) was added to the co-cultures. At the end of the incubation, supernatants were collected and total RNA or protein were extracted and immediately frozen at −80 °C for later analysis of different factors.

### 4.2. Sandwich Enzyme-Linked Immunosorbent Assay (ELISA)

The concentrations of endostatin, CD147, MMP-9, VEGF, Tsp-1, TIMP-1, and cathepsin S, as well as MMP-2, MMP-3, MMP-7, and MMP-14, were determined using the human DuoSet ELISA kits according to the manufacturer’s instructions (R&D systems). After calibration of each factor, serum samples or conditioned media were diluted at 1:100 for CD147, MMP-9 VEGF, TIMP-1, and MMP-2 in dilution buffer (1% BSA in PBS); 1:20 for endostatin; 1:250 for cathepsin S; and 1:1000 for Tsp-1.

### 4.3. Reducing CD147 Expression by siRNA

HT1080 cells (10^5^ cells) were reverse transfected with a mixture of two CD147-designed siRNA molecules (10 nM each) or with the siRNA negative control (NC, 10 nM) that were mixed with Lipofectamine RNAiMAX (Thermo Fisher Scientific, Waltham, MA, USA) diluted at 1:25 in Opti-MEM medium and incubated for 24 h in full medium without antibiotics to allow cell recovery. The medium was then replaced with serum-starvation medium supplemented with 0.1% BSA and TNFα (1 ng/mL) and cells were co-cultured for 48 h with U937 cells in inserts at a ratio of 1:1.

### 4.4. Wound Assay

The wound assay, assessing the angiogenic potential of cells, was described in detail in [21]. Briefly, a scratch was applied with a sterile tip to confluent EaHy926 endothelial cells and detached cells were washed away. The endothelial cells were then incubated with 100 μL of experimental conditioned media or with serum samples obtained from healthy volunteers and RA patients (both diluted 1:2 with the EaHy926 full medium), with and without the addition of the anti-CD147 (h161-pAb, 2 ng/mL). The inherent rate of gap closure was assessed by EaHy926 cells that received only full medium with no additions. Images of the scratch were acquired (Moticam 2MP, magnification ×4) immediately after the wound was applied (0 h) and at the end of the experiment (18 h or 20 h). Using the ImagePro plus 4.5 software (Media Cybernetics, Inc., Rockville, MD, USA), the distance between the two sides of the gap at 18 h was subtracted from the distance at 0 h to determine the average distance the endothelial cells proliferated/migrated to.

### 4.5. Tube Formation Assay

The 96-well plates were coated with the Coultrex^®^ reduced growth factor basement membrane extract (40 μL/well, R&D systems) at 4 °C and then allowed to polymerize for 2 h at 37 °C. Next, triplicates of the EaHy926 endothelial cells (8 × 10^4^ cells/well) were plated in DMEM with 2% FCS, together with supernatants obtained from either the co-cultures or the serum samples of RA patients that were diluted 1:2 with the medium. Images of the wells were captured after 6 h of incubation (Moticam 2MP, magnification ×4) and the closed lumens, representing two-dimensional tube-like structures, were counted in at least two separate fields.

### 4.6. Quantitative Real-Time PCR (qPCR)

Expression of Col18A mRNA was evaluated by qPCR, as described in [21]. One μg of total RNA extracted with the total RNA purification kit (Norgen Biotek, Thorold, ON, Canada) was transcribed to cDNA using the FIREScript RT cDNA synthesis mix (Solis bioDyne, Tartu, Estonia) at 37 °C for 1 h. The expression of Col18A1 mRNA and CD147 mRNA relative to their endogenous reference gene PBGD was determined by qPCR using the Eva-Green (Solis bioDyne) assay kit and the primers listed in Table S2. Additionally, 80 ng of cDNA were amplified for 40 cycles, each of 15 s at 95 °C and 60 s at 60 °C, in the StepOne real-time PCR (Thermo Fisher Scientific/Applied Biosystems, Waltham, MA, USA). The expression levels of miR-146a-5p and its endogenous reference RNU6B (U6) small RNA were reversed transcribed using the qScript microRNA cDNA synthesis kit (QuantaBio, Beverly, MA, USA) and 10 ng of the cDNA were amplified with the PerfeCTa SYBR green Supermix ROX (QuantaBio). The comparative method (2^−ΔΔCT^) for relative quantification was used and untreated cells served as calibrators.

### 4.7. Western Blot Analysis

ECM proteins that were deposited in the well were extracted using RIPA buffer and equal amounts (15 mg/lane) were loaded onto a 10% SDS-PAGE, separated, and transferred onto a nitrocellulose membrane (Advansta, San Jose, CA, USA). The membranes were blocked with Block-Chemi solution (Advansta) overnight at 4C and then incubated with the primary anti-endostatin antibody (R&D systems) diluted 1:3000 in blocking solution. After three washes with TBST (1× TBS with 0.05% Tween-20), membranes were incubated with the HRP-conjugated donkey anti-goat IgG (Jackson ImmunoResearch Labs, West Grove, PA, USA) diluted 1:5000 in blocking buffer, washed again, and incubated with Western Bright ECL (Advansta). Protein bands were visualized using the Omega Lum G imaging system (Aplegen, Pleasanton, CA, USA) and the density was quantified using the ImageJ 1.52a software.

### 4.8. Proteome Profiler Human Protease Array

Nitro-cellulose membranes arrived pre-spotted in duplicate with high-quality capture antibodies for multiplexing (R&D systems). The membranes were first blocked with the blocking buffer for 1 h. Then, diluted supernatants (1:2 with the array buffer) derived from co-cultures treated with or without tofacitinib (1 μM) were mixed with 15 μL of a mixture of anti-proteases detection antibodies and incubated for 1 h and, then, the mixture was added to the membranes for overnight incubation. After the incubation, the membranes were washed three times in the wash solution, followed by incubation with Streptavidin-HRP solution (diluted 1:2000) for 30 min. To detect signals, the chemiluminescent detection reagents were mixed (at a ratio of 1:1) and 1 mL was added to each membrane. All reagents were supplied in the kit. Membranes were exposed for up to 30 min in the Omega Lum G imaging system (Apelgen, Pleasanton, CA, USA). According to the instructions, the intensity of two spots for each protease in each membrane was averaged, the background was subtracted, and the ratio was determined. Each membrane has three pairs of reference spots that serve as an internal control.

### 4.9. MMP-9 Activity Assay

ELISA plates were coated overnight with the capture antibody for MMP-9 (2 μg/mL, R&D systems) to distinguish between the activity of MMP-2 and MMP-9 that cleave the same substrate. After washing the plate with PBS, 15 μL of supernatants derived from the co-cultures were incubated for 1 h. The wells were washed three times with PBS and the fluorescent substrate DNP-PLGMWSR (50 μM, trifluoroacetate salt, Cayman, Ann Arbor, MI, USA) was added in the assay buffer (50 mM Tricine, 0.2 M NaCl, 10 mM CaCl2, and 0.05% Brij-35) for 5 min. Then, the supernatant was transferred to a black 96-well plate, and the fluorescence released from the cleaved substrate was measured at 490 nm excitation and 520 nm emission. Recombinant MMP-9 that was activated overnight with Aminophenylmercuric Acetate (AMPA, 1 mM, Merck/Sigma-Aldrich, Darmstadt, Germany) was used as a positive control.

### 4.10. Proteasome 20S Activity Assay

In a black 96-well plate, supernatants (15 μL) derived from the co-cultures were diluted 1:8 with the proteasome 20S assay buffer (0.5M EDTA, 1M HEPES buffer, 1M DTT) and with the proteasome 20 s fluorescent substrate SUC-LLVY-2R110 (15 μM, AAT Bioquest, Sunnyvale, CA, USA) for 5 min. The cleaved peptide emitted fluorescence that was read at 490 nm excitation and 520 nm emission. The proteasome 20S preparation was activated for 1 h with 0.5% SDS and was used as a positive control.

### 4.11. Cathepsin S Activity Assay

Supernatants (10 μL) derived from the co-culture of HT1080 and U937 cells were diluted 1:5 with 1× PBS and incubated in a black 96-well plate, with 50 μL Cathepsin S reaction buffer (supplied in the kit) and 2 μL of cathepsin S fluorescent substrate Ac-VVR-AFC (200 μM, Abcam, Cambridge, UK), for 2 h. The fluorescence emitted by the cleaved peptide was read at 405 nm excitation and 500 nm emission. As a negative control, 1× PBS replaced cell supernatants.

### 4.12. Preparation of the De-Cellulararized Extracellular Matrix (ECM)

HT1080 cells (3 × 10^4^ cells/100 μL) were allowed to adhere in 96-well plates overnight in full medium. Then, the medium was replaced with HT1080 starvation medium with TNFα (1 ng/mL) and the cells were incubated for an additional 72 h to allow maximal deposition of ECM proteins. Then, supernatants were aspirated and cells were ruptured by a 20 min incubation in double distilled water (DDW). Cell debris was washed away three times in 1× PBS, leaving the de-cellularized ECM proteins, including Col18A, coating the well.

### 4.13. Study Population

Twenty RA patients with active disease, who met the 2010 American College of Rheumatology/European League Against Rheumatism (ACR/EULAR) RA criteria, were recruited from the rheumatology clinics in Carmel Medical Center and Bnai-Zion Medical Center in Haifa, Israel. Patients with a diagnosis of another inflammatory arthritis or neoplastic disease were excluded. Patients started treatment with 5 mg tofacitinib, taken orally twice a day along with conventional disease-modifying anti-rheumatic drugs (cDMARDs). Clinical and demographic data, including height, weight, calculated body mass index (BMI) (weight/height^2^), current medications, patient comorbidities, and data of physical examination focusing on swollen and tender joint counts, were collected at baseline (first visit, V1) and after one (second visit, V2), three (third visit, V3), and six months (fourth visit, V4). Laboratory parameters analyzed by standard methods at the central laboratory included complete blood count and chemistry panel, lipid panel, CRP, and rheumatoid factor (RF). These data are presented in Table 1. Serum samples were obtained for further analysis of the pro- and anti-angiogenic factors. Due to poor compliance and replacement of tofacitinib with other DMARDs at the discretion of the treating physician, only 14 patients completed 6 months (V4) of uninterrupted treatment.

Twenty-five healthy volunteers who displayed no inflammatory diseases, cardiovascular disease (CVD), renal failure, or any known malignancies and were gender-matched to the RA patients served as controls. This group was younger and the effect of age on the results could not be evaluated, rendering it a limitation. Blood samples from this group were drawn at recruitment.

### 4.14. Statistical Analyses

All values are presented as means ± SEM (standard error of the mean) and the number of biological repetitions (n) is specified in each figure legend. Two groups were compared using the unpaired two-tailed *t*-test. Multiple groups were compared using the one-way analysis of variance (ANOVA) followed by Bonferroni’s post hoc test and two parameters in multiple groups were compared using the two-way ANOVA and Bonferroni’s post hoc test. Patients’ data were analyzed using a two-tailed Mann-Whitney U test when two groups were compared; *p* values exceeding 0.05 were not considered significant.

## Figures and Tables

**Table 1:** Patient demographics, clinical characteristics, underlying diseases, and treatments.

	Control	RA Group	*p* Value	Non-Responding *	Responding *	*p* Value
Number of participants	25	20	-	3	11	
Sex: Female (%)	19 (95%)	17 (85%)	ns	3 (100%)	10 (91%)	ns
Age (years) ± SD	51.44 ± 2.10	65.1 ± 6.05	0.0007	80 ± 3.8	63.91 ± 3.7	0.0385
Body Mass Index (BMI) ± SD	26.56 ± 1.19	27.5 ± 1.09	ns	25.13 ± 2.5	27.47 ± 1.5	ns
Disease duration (years) ± SD	-	9.9 ± 1.9	-	22.0 ± 9.0	8.2 ± 2.01	ns
Tobacco use (%)	3 (15%)	4 (20%)	ns	0 (0%)	2 (18.2%)	ns
Alcohol use (%)	7 (35%)	0 (0%)	0.0123	0 (0%)	0 (0%)	ns
RF factor (%)	0 (0%)	17 (40%)	0.0017	1 (33.3%)	3 (27.3%)	ns
CRP ± SD	-	1.534 ± 0.33	-	0.29 ± 0.26	1.53 ± 0.43	0.0385
**Comorbidities**						
Hypertension (%)	-	10 (50%)	-	2 (66.6%)	7 (63.6%)	ns
Hyperlipidemia (%)	-	5 (25%)	-	1 (33.3%)	3 (27.3%)	ns
Diabetes mellitus (%)	-	4 (20%)	-	0 (0%)	3 (27.3%)	ns
**Medication (at baseline)**						
Methotrexate (MTX)	-	20 (100%)	-	3 (100%)	11 (100%)	ns
Leflunomide (LEF)	-	1 (5%)	-	0 (0%)	1 (9.1%)	ns
Sulfasalazine (SSZ)	-	1 (5%)	-	0 (0%)	1 (9.1%)	ns
Hydroxychloroquine (Plaquenil-PLQ)	-	3 (15%)	-	0 (0%)	3 (27.3%)	ns
Corticosteroid (%)	-	3 (15%)	-	0 (0%)	2 (18.2%)	ns

* The 14 patients that completed 6 months of treatment with tofacitinib were stratified according to their DAS28-CRP evaluation. ns, non-significant.

**Table 2:** Correlations of CD147 to serum factors and to disease severity scores in RA patients.

	Spearman r	95% CI	*p* Values
Endostatin	−0.1499	−0.469 to 0.203	ns
MMP-9	−0.0871	−0.423 to 0.268	ns
TIMP-1	0.6159	0.342 to 0.793	0.0001
CRP	0.7482	0.492 to 0.885	<0.0001
TJC	0.4659	0.136 to 0.703	0.0063
SJC	0.6656	0.409 to 0.825	<0.0001
CDAI	0.5259	0.186 to 0.753	0.0034
DAS28	0.5805	0.246 to 0.791	0.0015
MMP-9 activity	0.6529	0.395 to 0.815	<0.0001
Proteasome 20S activity	−0.6196	−0.796 to −0.347	<0.0001

## Data Availability

The original contributions presented in this study are included in the article and Supplementary Materials. Further inquiries can be directed to the corresponding authors.

## Supplementary Materials

The following supporting information can be downloaded at: https://www.mdpi.com/article/10.3390/ijms25137267/s1.
