# Supplementary material for: Tofacitinib Regulates Endostatin via Effects on CD147 and Cathepsin S
Source: Int J Mol Sci. 2024 Jul 2;25(13):7267. doi: 10.3390/ijms25137267 (PMC11241738; doi:10.3390/ijms25137267)
Supplement: Supplementary file 1 [file ijms-25-07267-s001.zip › ijms-3065600-supplementary.pdf]

Supplementary Materials

Table S1: Protease inhibitors used in this study

Inhibitor	Target/Specificity	Concentrations Used	Company
Pepstatin A	Aspartic acid proteases (e.g., cathepsin S, L, D, K)	5 μM	Merck
Leupeptin	Serine/cysteine/threonine proteases (e.g., cathepsin D, E)	1 μg	Merck
Phenanthroline	MMPs - wide range	40 μM	Merck
NSC 405020	Specific for MMP-14	100 μM	R&D systems
MMP-9 Inhibitor I	Specific for MMP-9	5 nM	Cayman
Disulfiram	Specific for both MMP-9 and Proteasome 20S	20 μM	R&D systems
MG-132	General for proteasome	1 μM	Merck
AM 114	Specific for proteasome 20S	1 μM	R&D systems

Table S2: Primers used for qPCR analysis

Gene	Fw sequence	Rv sequence
Col18A	5'-CACCACAGCTAGGTGCA	5'-CGCAACCAGGTGGAGCAC
CD147	5'CTGCCGGCAGTCTTCACTA	5'-CGTCCTCCTTCAGCACCAC
PBGD	5'-CAGTTTGAAATCATTGCTAT	5'-CTCCAATCTTAGAGAGTGCA
miR-146a-5p	5'-CCGAUGUGUAUCCUCAG	5'-UGAGAACUGAAUUCCAUG
RNU6B (U6)	5'-GCAAATTCGTGAAGCGTTCC	5'-UGAGAACUGAAUUCCAUG

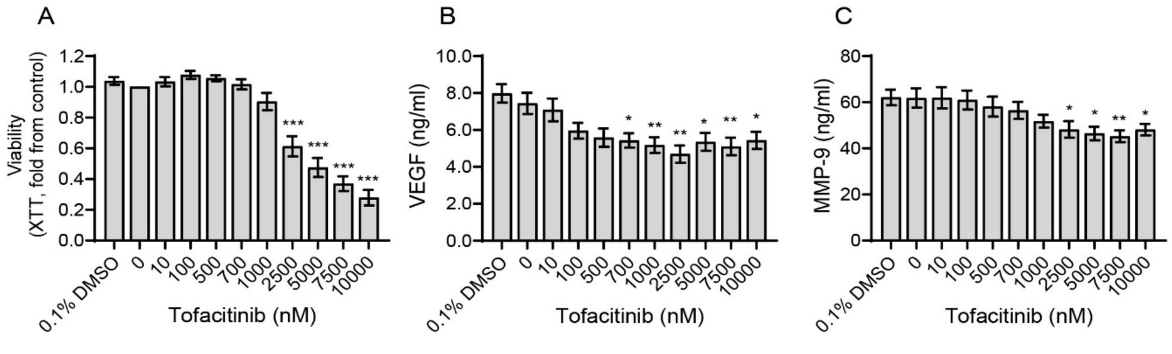

Figure S1: Calibration of tofacitinib concentrations. The HT1080 fibroblast cell line (3x10[4] cells) was incubated in co-culture with the U937 monocytic-like cell line (3x10[4] cells) for 48 in serum-starvation medium and in the presence of TNFα (1ng/ml) and different levels of tofacitinib. **(A)** Viability of the cells was determined using the XTT assay (n=13) and concentrations of **(B**) VEGF (n=13) and **(C)** MMP-9 (n=12) were determined in the supernatants using the ELISA. Data are presented as means ± SE and

were analyzed using one-way ANOVA followed by Dunnett's post hoc test.*, p<0.05, **, P<0.01, ***, p<0.001 relative to the untreated cells.

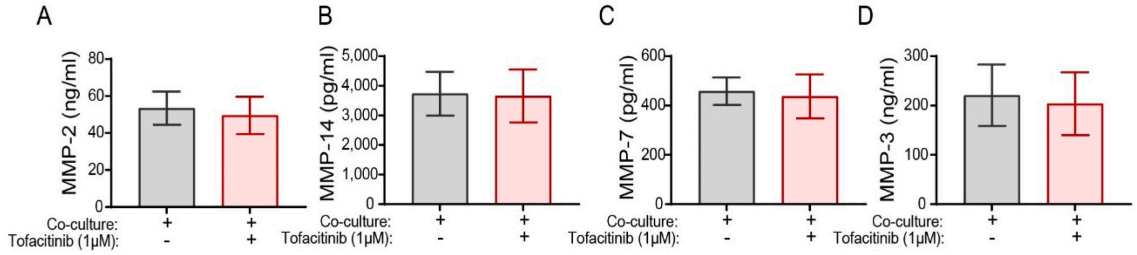

Figure S2: Tofacitinib does not affect MMPs other than MMP-9. Supernatants from co-cultures of HT1080 and U937 cells (see legend of Figure 1) were evaluated for the concentrations of other MMP family members that are associated with angiogenesis. **(A)** MMP-2, **(B)** MMP-14, **(C)** MMP-7, and **(D)** MMP-3 (n=12). Data are presented as means ± SE and were analyzed using the unpaired two-tailed *t*-test.

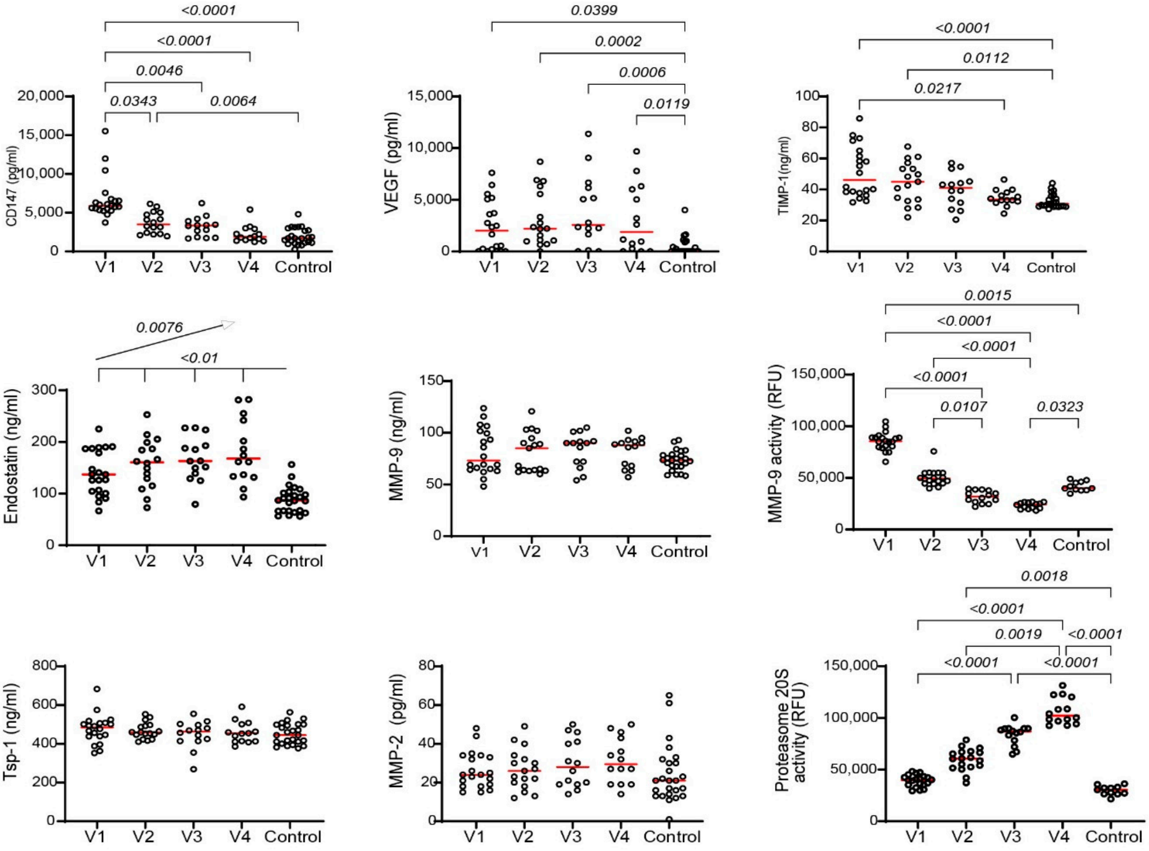

Figure S3: Tofacitinib affects pro- and anti-angiogenic factors in serum samples of RA patients over time. The concentrations of the angiogenic factors, as well as the activities of MMP-9 and proteasome 20S, were determined in serum samples collected from patients with active RA before the beginning of treatment (V1, n=20) and after one month (V2, n=16), three months (V3, n=14), and six months (V4, n=14)

of treatment with Tofacitinib. Serum samples from healthy volunteers (n=25) were used as controls. Data are presented as median values (red bar) and analyzed using the non-parametric Kruksal–Wallis ANOVA test followed by Dunn's multiple comparisons test.
